# Exploration of Urban Interaction Features Based on the Cyber Information Flow of Migrant Concern: A Case Study of China’s Main Urban Agglomerations

**DOI:** 10.3390/ijerph17124235

**Published:** 2020-06-13

**Authors:** Chun Li, Xingwu Duan

**Affiliations:** Institute of International Rivers and Eco-Security, Yunnan University, Kunming 650091, China; lichun@ynu.edu.cn

**Keywords:** urban interaction, information flow, urban agglomeration, urban network, cyberspace

## Abstract

In the context of “space of flow”, urban interaction has become the key force impacting urban landscape evolution and urban sustainable development. Current research on urban interaction analysis is mainly conducted based on the interaction of geographical elements, the virtual flow of information in cyberspace has not been given sufficient attention, particularly the information flows with explicit geographical meaning. Considering the dramatic population migration and the explosive growth of cyberspace in China’s main urban agglomerations, we constructed the information flow of migrant attention (IFMA) index to quantify the urban information interaction derived from public migrant concern in cyberspace. Under the framework coupling spatial pattern analysis and spatial network analysis, exploration spatial data analysis (ESDA) and complex network analysis (CNA) were adopted to identify the urban interaction features depicted by IFMA index in the three main urban agglomerations in China. The results demonstrated that, in the study area: (1) The IFMA index presented a reasonable performance in depicting geographical features of cities; (2) the inconformity between urban role in the network and development positioning confirmed by national planning existed; (3) in the context of New-type urbanization of China, urban interaction feature can be a beneficial reference for urban spatial reconstruction and urban life improvement. Using the cyber information flow with geographical meaning to analyze the urban interaction characteristics can extend the research angle of urban relationship exploration, and provide some suggestion for the adjustment of urban landscape planning.

## 1. Introduction

In the context of globalization and production fragmentation, cities are no longer islands and have begun to exploit scale economies in complementary relationships and synergies in cooperative activities. The pattern, which relies solely on internal know-how, does not fit in this development stage and brings the presence of “market failure” [1]. Under the continuous construction of high-speed interactive passages, intercity interactions expressed by the flowing of populations, materials, information, techniques, capital, etc., has been reinforced. This brings the achievement of urban economies of agglomeration [2]. The evolution pattern of urban space is transforming from the static “space of place” into the dynamic “space of flow” under the push of such intensified urban interaction [3]. Internal resources, environmental and socio-economic conditions are no longer the only force that impacts the change of urban landscape. External influences, accompanied by the activities of interaction between cities, merged into the process of urban development and promoted the form of urban organization mechanism in a networked and linked way. In such a context, the influence of urban interaction is further emphasized. Measuring and analyzing urban interaction feature becomes more and more crucial for effectively guiding urban landscape evolution, urban resource allocation, and protection of the urban environment, etc. [4,5,6,7].

Urban interaction represents the exchange of multiple regional elements, which cover various aspects of production activity and daily activities. It is the one thing that makes cities, which are spatially separated from each other, constitute an urban system with a certain structure and function. Existing studies regarding urban interaction can be divided into two types: studies based on the value chain or on the flow of elements. The value chain-based studies conventionally explore the characteristics of urban interaction from the perspective of the market. Based on a series of market activities, which aim at increasing the industrial value of the inner and outer city, the value chains network of urban areas, such as the allocation of global industry, the distribution pattern of headquarters and branches of multinational corporations, etc. are constructed [8,9,10,11,12]. This type of study conventionally explores urban interactions among global cities, mega cities, or regional core cities. The suitability of these studies for small cities or towns is limited due to the lack of industrial data. Another type of interaction research is conducted based on the monitoring and analysis of the flow of urban elements. This type of research can cover cities with different levels and sizes. They are based on the interaction links triggered by intercity elements flow that with orientation and quantitative features to conduct relevant exploration. The monitoring of actual flow and the modeling of flowing situations are the main approaches adopted. Thereby, the monitoring of actual flow can provide accurate and detailed information to explore urban interactions, such as the monitoring of cargo transport [13,14], population migration [15,16], intercity telecommunication flow [17], etc. However, the dynamic monitoring of actual flows required a great deal of time, manpower, and material resources to deploy, which may be too expensive for many researchers. Due to the difficulty in obtaining interaction data, many scholars have endeavored to derive models according to relevant theories, such as the theory of spatial interaction, the law of universal gravitation, and the field theory. A series of interaction models have been developed to help explore urban interaction features, including the Reilly model [18,19], the break point model [20,21], the Huff model [22,23], the maximum entropy model [24,25], the radiation model [26,27], the field-intensity model [28] etc. Along with the rise of the networked research framework, the exploration of element flow with the help of social network or complex network analysis gets more attention [29,30]. Through the construction of an urban network based on the modeling results of interaction models or actual interaction flows [31,32], relevant network indexes have been employed to identify the pattern and structure of urban interaction networks.

With the diversity and complications of urban systems, there are some deficiencies that should be supplemented in the current interaction research. In terms of the research objective, relevant studies mainly focused their attention on the urban interaction features depicted by the flow of entities elements in geographical space, the virtual elements flow, such as information flow in cyberspace, has not been well studied. Some studies concerned with the flow of insubstantial elements mainly conduct their analyses based on the interaction structure of the element itself [33]. The explanation of the actual meanings of such flows is insufficient. From the angle of the research approach, relevant research largely focuses on the topology structure analysis of urban interactions. The comprehensive analysis of urban distribution patterns coupled with interaction structures is deficient. As we know, different elements’ interactions typically provide distinct urban characteristics, and the lack of exploration regarding virtual elements flow may pose some impediment to the comprehensively understanding of urban development in the current information era. Both the spatial distribution pattern and interaction relationship of urban elements are important in pushing the networked development of urban areas, the comprehensive analysis is necessary.

Urban agglomeration is the new spatial unit that currently participated in global competitions that is also the organic whole with complex and close inner interactions [34]. The exploration of urban interaction features is extremely important in this area, especially for China’s urban agglomerations. Since the reform up in 1978, China has experienced rapid urbanization and economic growth [35]. The enormous and widespread flowing of urban elements can be identified in such processes. Therein, one of the most significant flow can be seen as the migration of population. In 2015, there was an estimated population of 247 million migrants in the whole country, which accounted for 17.96% of the total national population [36]. Meanwhile, along with the explosive growth of cyberspace, the flow of information has become more and more convenient in China. Taking the amount of netizens as an example, the number has increased from 4 million in 1999 to 688 million in 2015, and the internet penetration rate (the ratio of netizens to the total population) in 2015 reached 50.30% [37,38]. Such dramatic flows of regional elements have reinforced urban interactions and have pushed the construction of urban networks in China, both in geographical space and cyberspace. The role of urban agglomeration in this process cannot be neglected [34]. Taking the three national urban agglomerations in China [39] as an example, they occupy only 5.09% of national territory but have the highest urbanization rate. In 2015, the amount of the migrant population in these areas has arrived more than 65 million, accounting for 25.32% of the total migrants [36]. Internet penetration has reached more than 70%, which was much higher than that of the whole country [37]. These areas had the most active urban interaction and are leading the construction of urban network in China. Therefore, understanding and exploring urban interaction features in the urban agglomerations of China can provide valuable information for improving inner urban relationships, nurturing urban systems, and realizing regional coordinated development. It is a good reference not only for China but also for other regions where with dramatic interactions resulted from elements’ flows.

In this paper, focused on the increasing influence of urban interaction under networked develop trends of urban space, we aim at exploring the urban interaction characteristic through the analysis of information flows. Considering the widely observed population migration and the explosive growth of cyberspace, the cyber information flow, which has not received enough attention in relevant studies but occupied a significant role in the current information era, is quantified to construct an information flow of migrant attention (IFMA) index. Migration-related cyber search queries have been adopted in IFMA index to express intercity information flow triggered by public migrant concerns in cyberspace. Taking three main urban agglomerations of China as study area, the comprehensive framework coupling spatial pattern description and spatial network analysis is built to explore urban interaction features conveyed by IFMA index in these areas. Based on the research, we strive to extend the research angle of urban interaction, combine the geographical meaning with virtual information flow, and provide some support for healthy management and planning of urban areas. This paper is organized as follows: In Section 2, we introduce the study area and data. In Section 3, we present the methods used in this paper, including the construction of the IFMA index and the analysis framework that couples spatial patterns and spatial networks. Section 4 reports the results from the spatial characteristic analysis. Section 5 conducts the further discussions based on the results. Last, in Section 6, we conclude this paper.

## 2. Study Area and Data

### 2.1. Study Area

**Figure 1:**
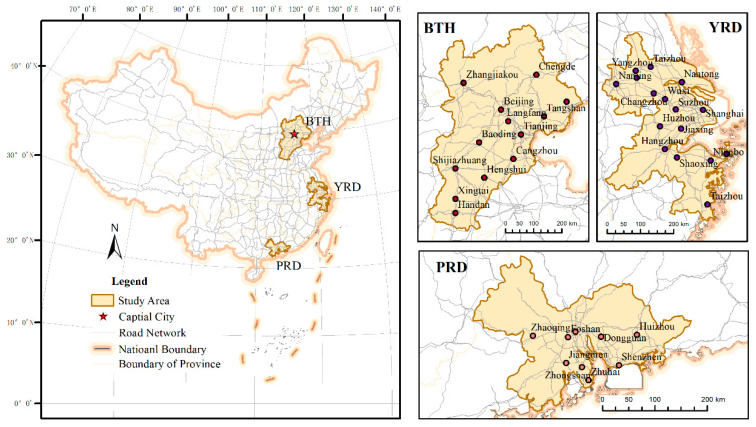
Locations of the study area.

The Chinese urban spatial structure is composed of three metropolitan regions and 13 urban agglomerations [40]. Therein, the Beijing-Tianjin-Hebei metropolitan region (BTH), the Yangtze River Delta (YRD), and the Pearl River Delta (PRD) are the three world-class metropolitan interlocking regions of China. In this study, we selected these three metropolitan regions, which included 38 cities (i.e., 13 cities from BTH, 16 from YRD, and nine from PRD), as our study area, as shown in Figure 1. According to the Nationwide Urban System Planning of China (2006–2020), there are four national central cities (i.e., Beijing, Tianjin, Shanghai, and Guangzhou), and five regional central cities (i.e., Shijiazhuang, Shenzhen, Nanjing, Hangzhou, and Ningbo) located in the study area. Remarkable advantages in scientific and technological innovations, transportation, international competition, cultural influence, human resources, etc. can be observed in these areas and bring them a powerful attractiveness. From the perspective of urban element distribution, this study area aggregates massive urban resources. For instance, these areas occupy approximately 5.09% of the land of China, but they accounted for 23.65% and 39.87% of the national permanent residents’ and gross domestic product (GDP) in 2015, respectively. From the perspective of urban network links, these regions are significantly superior in the layout of interaction corridors, such as the wide distribution of modern ports and airports, well-organized road networks, and integrated regional policies, etc. Dominant roles in promoting the development of the national urban system can be depicted in these regions. Therefore, research focused on these areas can be a good reference not only for guiding the healthy development of China’s urban system but also for exploring urban interaction features in other urban areas with drastic elements flows.

The consistent importance of these regions in China’s development has been confirmed by national planning, which shows that they not only play supporting roles in constructing the multi-center pattern of China’s urban system but also be appointed as the core areas of China to participate in the global economic competition in the future [39]. Thus, exploring these areas is also necessary for implementing national general planning and improving urban competitiveness in future China.

### 2.2. Data

The data mainly used in this paper included the dynamic monitoring of migration data, the basic geographical information data, and the search query data in cyberspace. The dynamic monitoring data of migration were used to confirm the major reasons causing public attention on migration in cyberspace. It is obtained from the dynamic monitoring survey of China’s migration population in 2015 (http://www.chinaldrk.org.cn/wjw/#/home), which was conducted by the Migrant Population Service Center attached to the National Health Commission of China. Meanwhile, the basic geographic information was obtained from the National Geomatic Center of China. Finally, search query data were collected based on the Baidu Index-offered by the Baidu search engine which is a widely used internet tool and provides convenient and low-cost information for internet users. To better exhibit and exploit the search query data, relevant search exploit services based on search query data were produced, typically as Google Trend (www.google.com/trends/) and Baidu Index (http://index.baidu.com/). In 2015, there were 566 million search engine users in China, accounting for 82.27% of the national netizens. A series of studies have been conducted that analyzed data from Google Trend and Baidu Index, and their robustness and effectiveness were assessed [41,42,43,44]. In China, compared to Google, which is the largest search engine in the world, Baidu has a larger share (77.07%) of the internet search engine market [37]. Vaughan and Chen [45] collected and compared the data from Google and Baidu, and found that Baidu Index can offer more search volume data than Google Trend in China. In this context, Baidu Index was employed to quantify search queries in the cyberspace.

## 3. Methodology

**Figure 2:**
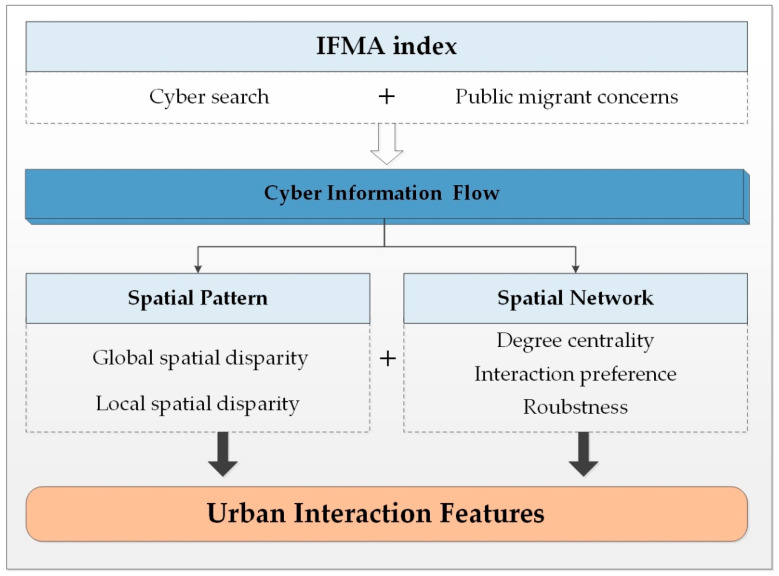
Research framework.

Urban interaction as the significant force for urban evolution in the context of rapid element flowing, exploring its feature has become the inevitable content for identifying urban develop trends and achieving the agglomeration economics of cities. Conventional researches often explore urban interaction features based on the entity elements in geographical space, the interaction features expressed by massive information flow in cyberspace have been neglected. In this paper, we endeavor to explore the characteristic of urban interaction based on the flowing of information in cyberspace, and further support the healthy development of urban space in the process of networked evolution. To identify such interaction features, two issues should be concerned: first, how to quantify the information flow with geographical meaning in cyberspace; second, how to identify the characteristic of urban interaction based on such cyber information flow. For the first issue, the information flow of migrant attention (IFMA) index, which based on the public attention on migrating to specific cities in cyberspace, has been constructed to quantify the information flow with objective geographical meaning. For the second issue, the analysis frameworks of spatial pattern and spatial network have been coupled. Employing the approach of exploratory spatial data analysis (ESDA) and complex network analysis (CNA), we explore the comprehensive features of urban interaction from the perspective of spatial distribution and interactional links. The research framework is displayed in Figure 2.

### 3.1. The Expression of Information Flow Derived from Public Migrant Concerns in Cyberspace

To measure cyber information flow with geographical meaning which indicated population migration, three issues should be investigated: (1) What causes the public attention on migration? (2) How the causes of migration in cyberspace be delineated? (3) How to comprehensively express the information flow triggered by public migrant concerns?

**Figure 3:**
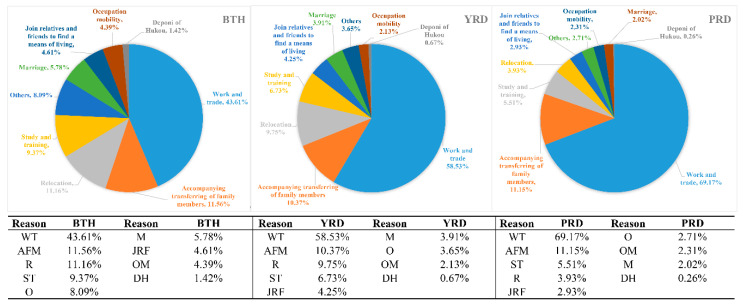
The percentage of migrant population based on diverse migration reason in the study area. WT: Work and trade; AFM: Accompanying transferring of family members; R: Relocation; ST: Study and training; O: Others; M: Marriage; JRF: Join relatives and friends to find a means of living; OM: Occupation mobility; DH: Deponi of Hukou.

For the first issue, we adopted the results from the dynamic monitoring survey of China’s migration population in 2015 as a reference, based on the statistics of the major reasons for migration in the three urban agglomerations to confirm the major causes in evoking public attention on migration, as shown in Figure 3. It can be seen that work and trade, study and training, accompanying transferring of family members and relocation were the main migration factors in the study area. As the transferring of family members always accompanies family relocation [46], we viewed them as one perspective and marked them as relocation. Therefore, three main reasons for population migration were confirmed as work and trade, study and training, and relocation.

For the second issue, in the internet era, the application of cyber tools can help internet users to promptly acquire low-cost information. The flowing of information triggered by individual action in cyberspace has become more and more convenient. As the most widely used internet tools, search engines provide information and also record individual attention on specific objectives. The collection of public concerns on specific search keywords can quantify the interaction of information which with certain directions in cyberspace. In this paper, we selected a series of migration-related keywords, which are suited for cyber searching, to depict the public attention on migration derived from distinct migrant reasons. The selection of keywords following the least effort principle in cyber information retrieval behaviors [47,48], the basic structure of keywords is set as in Chinese, with simple and brief features. The specific keyword candidates were confirmed by brainstorming common words used in searching for migration and review of related literature [49,50,51,52]. Words related to the keyword candidates were also been selected, such as “rent” and “lease”.

For the third issue, the information flow of migrant attention (IFMA) index was constructed to comprehensively depict the information flow derived from public migrant attention in cyberspace. The construction of the IFMA index followed four steps: (1) The keywords candidates and their similar words were combined with the names of objective cities to construct the combined search keywords, which express public migrant attention on the objective cities. (2) The Baidu Index (BI) offered by the Baidu search engine was employed to quantify the cyber attention on the combined search keywords during a specific duration of time, to identify the intercity information flow with distinct original and terminal cities. (3) The sifting of keywords was conducted following the principle of maximum value and continuity in time. The most popular keyword was chosen from a series of its similar words (e.g., “house renting” with BI: 11,795 was selected, “rent” with BI: 477 and “lease” with BI: 636 were removed), keywords without a continuous BI in a year resolution are also removed. The retained of combined the combined keywords were ensured to be the most popular and effective ones. Then, the correlations among them were tested to reduce data redundancy. The final qualified keywords were confirmed as shown in Table 1. (4) Based on the qualified search keywords, the IFMA index was constructed to comprehensively express the information flow triggered by migrant attention in cyberspace. The formula can be seen in Equations (1) and (2).

(1) $$IFMA_{i}=\sum_{j=1}\sum_{n=1}^{3}W_{ijn}\times IF_{n}/IFMA_{\max},i\neq j$$

(2) $$IFMA_{ij}=\sum_{n=1}^{3}W_{ijn}\times IF_{n}/IFMA_{\max},i\neq j$$

where, *IFMA_i_* is the total amount of cyber migrant concerns that city *i* received from urban external areas; *IFMA_ij_* is the information flow triggered by migrant attention derived from city *j* to city *i* in cyberspace; *IF_n_* is the average value of Baidu Index regarding different search keywords under migration reason *n*; *W_ijn_* refers to the weights of *IF_n_*, which are defined by the proportion of people who migrate into city *i* for this reason; and *IFMA*_max_ is the maximum absolute value of the IFMA indexes.

### 3.2. Interaction Characteristic Analysis of Urban Area Based on the Cyber Information Flow

#### 3.2.1. Spatial Pattern Characteristics

To investigate the spatial distribution characteristics of IFMA, we introduced exploratory spatial data analysis (ESDA) to visually describe the *IFMA_i_* of a single city in an effort to identify regional problems. ESDA combined statistical analysis with geospatial information to explore the aggregation and dispersion characteristics in geographical space [53]. In this paper, Moran’s I and local indicators of spatial association (LISA) were utilized to analyze the spatial adjacency relationship and the spatial pattern of regional units depicted by *IFMA_i_*. The global Moran’s I reflects the comprehensive situation of the whole area [54], which can be used to express the holistic characteristic of the spatial pattern presented by *IFMA_i_*. The formula of Moran’s I can be expressed as:

(3) $$I=\frac{1}{\sum_{i=1}^{n}\sum_{j=1}^{n}w_{ij}}\times \frac{\sum_{i=1}^{n}\sum_{j=1}^{n}w_{ij}\left(x_{i}-\bar{x}\right)}{\sum_{i=1}^{n}{\left(x_{i}-\bar{x}\right)}^{2}/n},i\neq j$$

where, *I* is the regional Moran’s I index, *x_i_* is the observed *IFMA_i_* value of unit *i*; *x* is the average value of *IFMA_i_*, and *w_ij_* is the binary spatial weight matrix created based on the distance standard to reflect the locational similarity between spatial objects.

LISA can further explore the spatial heterogeneity which exists in the partial area, it will offer powerful help in confirming the high-impact areas in the specific region [55]. The formula of LISA is shown as:

(4) $$LISA_{i}=Z_{i}\sum_{i=1}^{n}w_{ij}Z_{j}$$

where, *LISA_i_* is the LISA index of spatial unit *i*, *Z_i_,* and *Z_j_* are the standardized results of objects *i* and *j*’s observed *IFMA_i_* values.

The Z test was employed to test the significance of Moran’s I and LISA. Critical values of 1.96 and −1.96, which through the two-sided significance test in 95% confidence interval, were viewed as the standard to judge the significance. The formula can be shown as follows:

(5) $$Z(I)=\frac{1-E(I)}{\sqrt{Var(I)}}$$

where, *E*(*I*) shows the expectation value of the objective index, and *Var*(*I*) is the variance of the objective index.

#### 3.2.2. Spatial Network Characteristic

Based on the definition of the IFMA index, *IFMA_ij_* is that index displays the information flow among cities. It can be used to delineate the quantity of information flow among cities with different scales and administrative levels, which also can indicate the derived and targeted direction of information flow. In this paper, an urban interaction network triggered by cyber information flow was constructed based on the *IFMA_ij_* index. A sequence of networked indicators was employed to analyze the characteristics of such networks.

(1) The Construction of nIFMA

To further delineate the spatial features of information flow, we constructed the spatial network of *IFMA_ij_* (nIFMA) to help express the interaction characteristics of networked cities. Urban nodes were viewed as network nodes, and the information flow depicted by *IFMA_ij_* between different cities was viewed as network links. To eliminate the negative influence from spurious and statistically insignificant links for analysis and visualization of the urban network, we adopted the global threshold method to extract the major structure of nIFMA under the principle of no harm for urban network connectivity. Two strategies were adopted to analyze and spatially explicit nIFMA: the geospatial location-based approach and the graph theory-based approach. Therein, the geospatial location-based approach expresses urban network based on the geospatial location of urban nodes, urban network can be depicted comprehensively by coupling the networked information with the geographical distribution. The graph theory-based approach was then implemented by adopting the Fruchterman-Reingold force model [56], urban networks can be spatially explicated in a relatively symmetrical and balanced way.

(2) Network Structure Analysis

Network indexes from complex network analysis are effective tools to reflect the interaction characteristics of a specific network. A series of network indicators were employed to explore the features of nIFMA. From the network, node, and edge scales, eight network analysis indicators were confirmed, including the degree, weighted degree, network diameter, graph density, number of nodes, average clustering coefficient, number of edges, and average path length.

Especially, the degree centrality of network nodes was estimated to analyze the interaction role of each city in nIFMA. Regarding the directed property of the *IFMA_ij_* network, two kinds of centrality indexes were employed to delineate the urban network features in diverse directions: In-degree centrality and out-degree centrality. Focused on the urban attractiveness for cyber attention, we adopted in-degree centrality to represent the urban positive influences for the inflow of information, and the formula can be shown as follows:

(6) $$D_{I\_i}=\sum_{j=n}a_{ij}$$

where, *D_I_i_* is the in-degree centrality of city *i*, and *a_ij_* is the number of network links that originate from city *j* to target city *i*.

Furthermore, the degree correlation was adopted to investigate the urban interaction preference in nIFMA. It is the relationship between cities’ degree *k* and the average degree of the neighbors of all *k*-degree nodes [57]. Cities with a high degree of centrality incline to interact with each other is referred to as assortativity. Otherwise, cities with a high degree of centrality incline to interact with low-degree nodes is referred to as disassortativity [58]. The average degree of each urban node can be defined as:

(7) $$k(i)=\frac{1}{k_{i}}\sum_{j\in N_{i}}k_{j}$$

where *k*(*i*) is the average degree of *N_i_*, *k_i_* and *k_j_* are the degree of city *i* and *j* respectively; and *N_i_* is the number of urban nodes that link with city *i*. The average degree of all k-degree nodes’ neighbors can be defined as:

(8) $$\bar{K(k)}=\frac{1}{N(k)}\sum K(i)$$

where *N*(*k*) is the number of urban nodes with the degree of *k*. The degree correlation is the relationship between *k* and $\bar{K(k)}$.

To effectively describe how the urban network reacts to external attacks [59], we evaluated the robustness of nIFMA. The largest connected component size F, which was a fraction of the network size, was used in quantitatively measuring the robustness of the urban network. Following different urban nodes removal strategies, including the removal of none cities, national central cities, regional central cities and others, the largest connected component size was measured to analyze the network robustness and the urban roles in the urban network of cyber information flow.

## 4. Results

### 4.1. Size and Ranking of IFMA_i_

**Figure 4:**
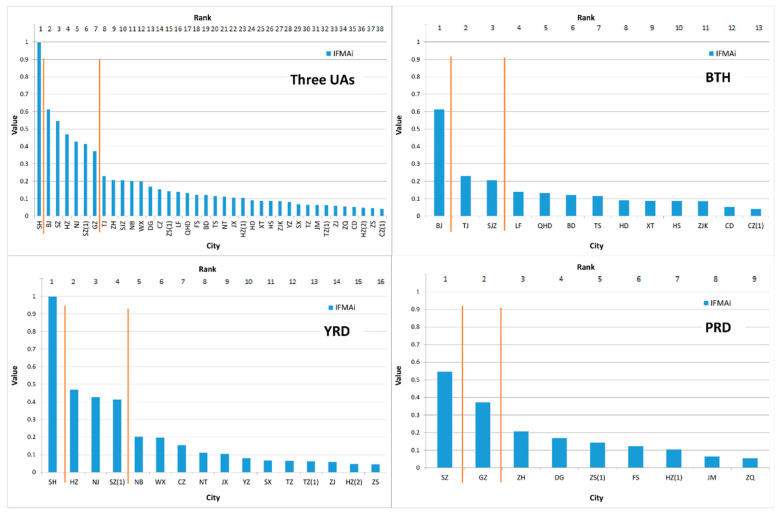
Rank and size of *IFMA_i_.* SH: Shanghai, BJ: Beijing, SZ: Shenzhen, HZ: Hangzhou, NJ: Nanjing, SZ(1): Suzhou, GZ: Guangzhou, TJ: Tianjin, ZH: Zhuhai, SJZ: Shijiazhuang, NB: Ningbo, WX: Wuxi, DG: Dongguan, CZ: Changzhou, ZS(1): Zhongshan, LF: Langfang, QHD: Qinhuangdao, FS: Foshan, BD: Baoding, TS: Tangshan, NT: Nantong, JX: Jiaxing, HZ(1): Huizhou, HD: Handan, XT: Xingtai, HS: Hengshui, ZJK: Zhangjiakou, YZ: Yangzhou, SX: Shaoxing, TZ: Taizhou, JM: Jiangmen, TZ(1): Taizhou, ZJ: Zhenjiang, ZQ: Zhaoqing, CD: Chengde, HZ(2): Huzhou, ZS: Zhoushan, CZ(1): Cangzhou.

The size and rank of the 38 cities’ *IFMA_i_* are depicted in Figure 4. It can be observed that Beijing, Shanghai, and Shenzhen, which separately role as the predominant cities in their own urban agglomerations, are the top three cites in attracting public migrant attention in cyberspace. Among all 38 cities, the values of the top 12 cities were higher than the average of the *IFMA_i_* indexes. Therein, three cities (i.e., Beijing, Tianjin, and Shijiazhuang) located in the BTH (north China), six cities(i.e., Shanghai, Hangzhou, Nanjing, Suzhou, Ningbo, and Wuxi) located in the YRD (eastern China) and another three cities(i.e., Shenzhen, Guangzhou, and Zhuhai) located in the PRD (south China).

In the three urban agglomeration areas, two breakpoints could be separately found in their interior domains. In the BTH, the first breakpoint lies between Beijing and Tianjin. The *IFMA_i_* of Beijing was 165.65% higher than that of Tianjin. Significant deviance can be observed to emphasize the predominant role of Beijing in the BTH; in the YRD, a similar situation can be seen between Shanghai and the second ladder cities (the *IFMA_i_* of Shanghai was 112.38% higher than that of Hangzhou), while a slightly different pattern was exhibited in the PRD. Two break points partitioned Shenzhen, Guangdong and other cities into three parts. The deviance between the first and second ladder (46.50%) was relatively lower than that of the BTH and the YRD, and two leading cities were detected in the PRD.

Comparing the urban size depicted by *IFMA_i_* with the urban roles classified by regional planning, it can be seen that, the national and regional central cities conventionally displayed a relatively higher interaction size in the study area. However, some incongruity was also observed. For the national central cities, the interaction size of Tianjin and Guangzhou were obviously insufficient, their *IFMA_i_* even lower than that of three regional central cities and one non-central city.

For the regional central cities, the interaction sizes of Shijiazhuang and Ningbo were relatively lower than the expected value of the regional planning. For the non-central cities, Suzhou and Zhuhai deserve more attention. Especially the Suzhou City, its interaction size depicted by *IFMA_i_*, surpassed the national central cities—Guangzhou and Tianjin.

### 4.2. Spatial Pattern of IFMA_i_

#### 4.2.1. Global Spatial Disparity

**Figure 5:**
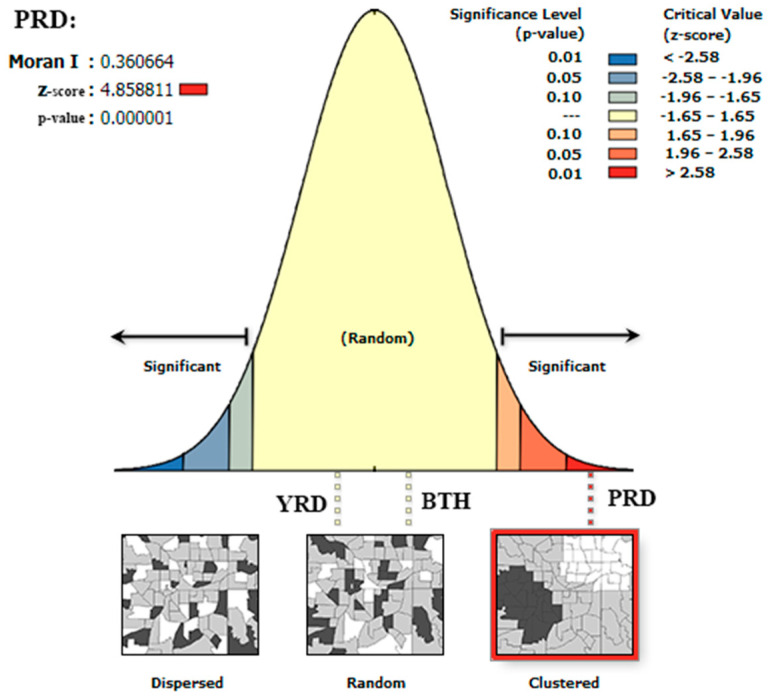
The distribution pattern of *IFMA_i_* in the different urban agglomerations.

To analyze the spatial correlation of *IFMA_i_*, the Global Moran’s I of *IFMA_i_* in the BTH, the YRD, and the PRD were separately calculated; then the Z test was employed to test the significance of Moran’s I. The results are shown in Table 2. Only the PRD with a *Z*-score of 4.86, which was lower than the critical value of the two sides test of normal distribution at 95% confidence interval (−1.96), had a significant Moran’s I index. This indicates that the *IFMA_i_* of the cities in the PRD had a clustered trend in geographical space, as shown in Figure 5. The cities with similar *IFMA_i_* characteristic incline to represent aggregated distribution in the PRD. 

#### 4.2.2. Local Spatial Disparity

**Figure 6:**
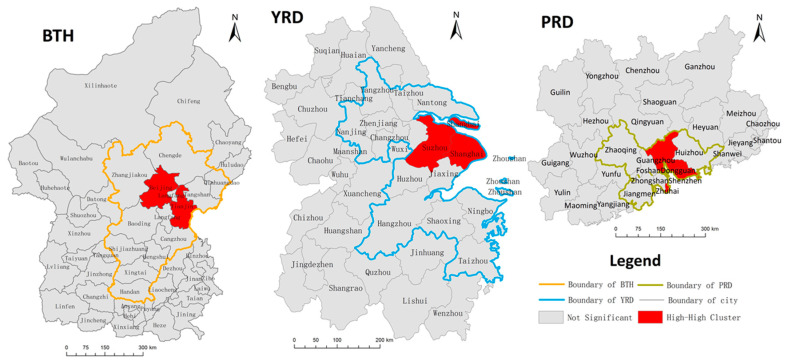
The local indicators of spatial association (LISA) of *IFMA_i_* in the different urban agglomerations.

To further explore the spatial association patterns in local areas, we analyzed the LISA index of *IFMA_i_* and tested their significance in an extensional spatial scope. The results were spatially explicit expressed in Figure 6.

Most of the cities did not have a significant spatial correlation locally, except for the east of BTH (i.e., Beijing and Tianjin), the east of YRD (i.e., Shanghai and Suzhou) and the south of PRD (i.e., Guangzhou and Shenzhen). The type of spatial correlation patterns in the study area were mainly High-High clusters, which demonstrated that the spatial concentration was mainly observed among the cities with a high *IFMA_i_*. The spatial location of the cluster cities coincided with the areas with significant spatial correlations. For individual cities, the High-High cluster cities all had noticeable influences in the region. In the BTH, Beijing and Tianjin were the only two national central cities, and a large number of resources were gathered in these two cities; the same situation was observed in PRD, where Guangzhou and Shenzhen are separately the national central city and the regional central city, similarly gathered with enormous regional resources; in the YRD, although Suzhou is not the central city assigned by the national plan, it has a substantial population and economic scales, even larger than the provincial capital of Jiangsu Province—Nanjing.

### 4.3. Spatial Network Analysis of nIFMA

**Figure 7:**
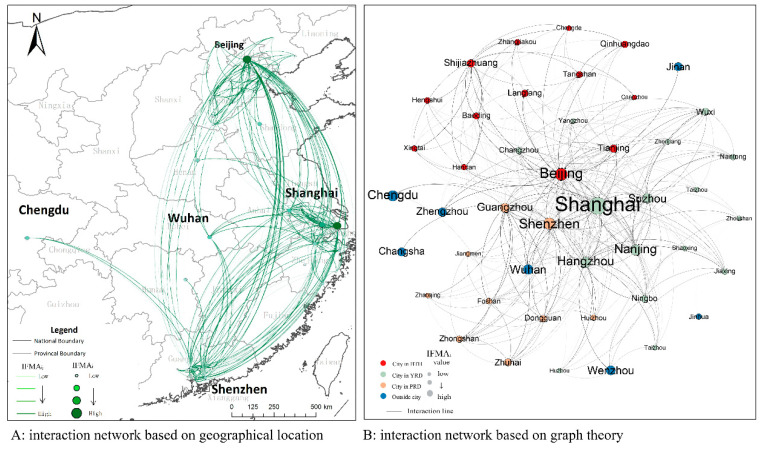
The network of *IFMA_ij_*.

We constructed the network of *IFMA_ij_* (nIFMA), the 38 cities and the cities most interested for them in the top 10 have been viewed as nodes in the network. In addition to the 38 cities, another seven cities were selected, including Jinan, Zhengzhou, Wuhan, Changsha, Chengdu, Wenzhou, and Jinhua. The cyber information flows of public migrant attention between the different cities were viewed as links. There were 342 links have been finally built in the network. The Baidu Index of the given migration keywords and the city’s name was collected to calculate the *IFMA_ij_*. The *IFMA_ij_* from city *j* to city *i* was defined as the weight of link*_ij_*. Based on the geographical location of each city, we expressed the network in a spatially explicit way. Simultaneously, the Fruchterman-Reingold force model was employed to establish the network from the perspective of graph theory, as shown in Figure 7. Under the assistance of complex network analysis, the main measured indexes of the whole network were obtained as demonstrated in Table 3.

A random network of the same size was constructed for comparison. The average clustering coefficient and the average path length of the random network were separately 0.178 and 2.06, respectively. A higher clustering coefficient and a shorter path length depicted the small-world features of nIFMA. Though the combination of the two types of networks, we can see that the network can be distinctly divided into three parts. Except for a small number of cities located in the middle of the network, most cities paid more attention to the internal cities of its attached agglomeration. For example, Shijiazhuang, which is located in the BTH, was more likely to concern the cities located in the BTH than the other two urban agglomerations; Zhongshan, which is located in the PRD, more likely to concern the cities located in the PRD. A lack of interregional migrant attention was observed in most objective cities. The noticeable role of geographical distance in affecting individuals’ migrant attention could be revealed from such spatial network pattern.

Beijing, Shanghai and Shenzhen occupied the core state in the network, which could be delineated as the hubs of the network. Their prominent role in obtaining the influx of cyber information can be further displayed. In the periphery, they were separately followed by Tianjin in the BTH, Suzhou, Nanjing and Hangzhou in the YRD and Guangdong in the PRD. Stronger support from other cities for the predominant city was depicted in the YRD. It is noteworthy that Shijiazhuang, as a regional central city in the BTH area, had an obvious marginalized location in the network compared to other regional central cities, even more marginalized than the regional regular cities. This highlights that the network function of Shijiazhuang, whose role of a regional center, was not well demonstrated.

#### 4.3.1. Degree Centrality of Cities

**Figure 8:**
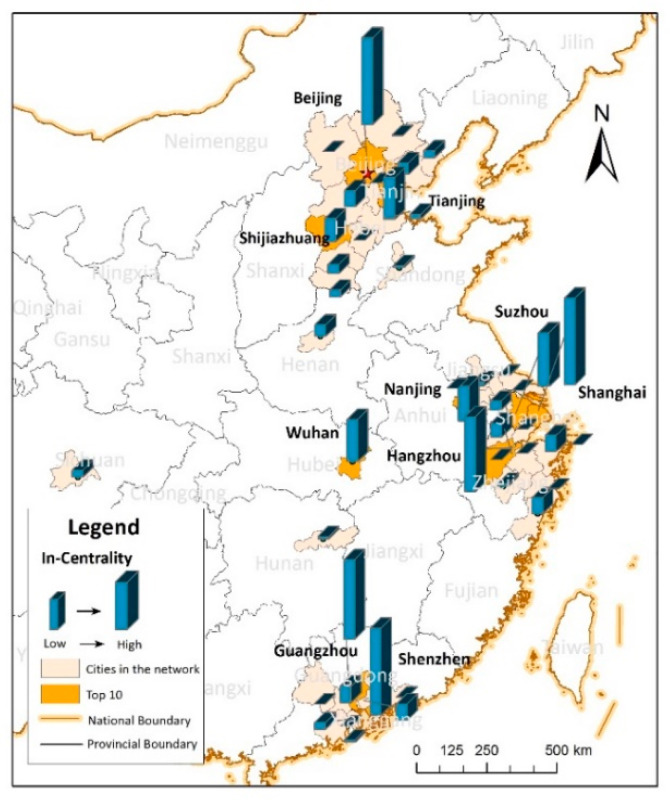
Spatial distribution of urban in-degree of centrality.

Based on Equation (6), we calculated the in-degree centrality of each city, and the results are spatially expressed in Figure 8. Beijing, Shanghai, and Shenzhen had the highest in-degree centrality in the network, which implies that they were the most concerned cities in nIFMA. There were 10 cities with an in-degree centrality higher than the average value: Beijing, Shenzhen, Shanghai, Guangzhou, Hangzhou, Suzhou, Tianjin, Wuhan, Nanjing, and Shijiazhuang. We further analyzed the characteristics of these 10 cities and found that they also had the population scales larger than the average of the 38 cities, and they were listed in the top 30% of the population scale rank. Their economic scales were also ranked in the forefront. Except for Shijiazhuang, all the nine cities with GDP larger than the average value and ranked in the top 24% of all cities. Considering the hierarchy of these cities, which released by Nationwide Urban System Planning of China 2006–2020 (the planning generally states the specific function of cities based on their composite influence in the urban system of China), there were four national central cities and five regional central cities. A relatively higher attractiveness of the central cities was displayed compared to the regular cities. The degree centrality characteristics depicted by *IFMA_ij_* essentially matched the situation of the urban population, the economic and urban status in regional planning.

#### 4.3.2. Interaction Preference of Cities

**Figure 9:**
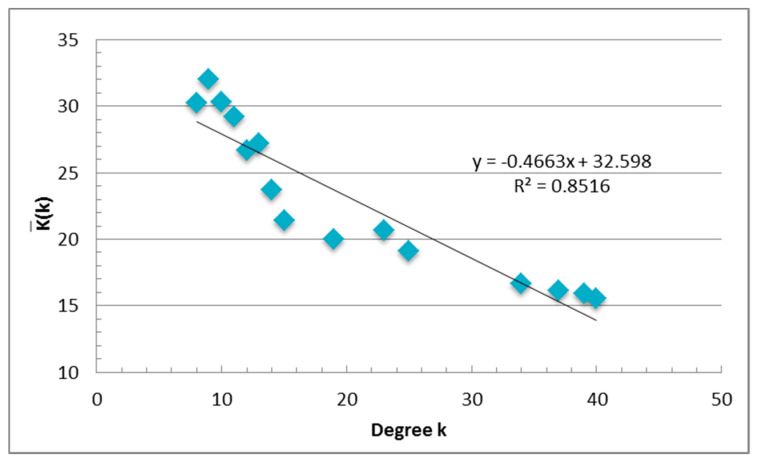
The degree correlation of nIFMA.

To investigate the interaction preference of the objective cities, based on Equations (7) and (8), we calculated degree correlation of nIFMA as −0.4663, which follows a linear function: y = −0.4663x + 32.598 (R2 = 0.8516), as shown in Figure 9. The results delineate the disassortativity in the degree correlation. The higher the degree of centrality the node owns, the lower the average degree owned by its neighbors. For instance, Beijing had the highest degree of 40 and the lowest neighbors’ average degree of 15.55. Shenzhen and Shanghai had the second highest degrees of 39, with the neighbors’ average degrees of 15.90 and 15.92, respectively. Among the low-degree cities, the cities with a degree of 9 had the highest K(k) as 32.02, and the cities with degrees of 10 and 8 ranked the second and third highest K(k). The disassortativity in the low degree cities was more considerable, when k ≤ 15, with y = −1.1715x + 41.383 (R2 = 0.8167); and when the k ≥ 15, with y = −0.2395x + 25.123 (R2 = 0.9605). The results show that the low-degree cities preferred the high-degree cities more than the preference of high-degree cities for low-degree cities. For the individual cities, the national and regional central cities had a relatively lower K(k). The higher attractiveness of these cities for regional regular cities can thus be delineated.

#### 4.3.3. Robustness of the Interaction Network

**Figure 10:**
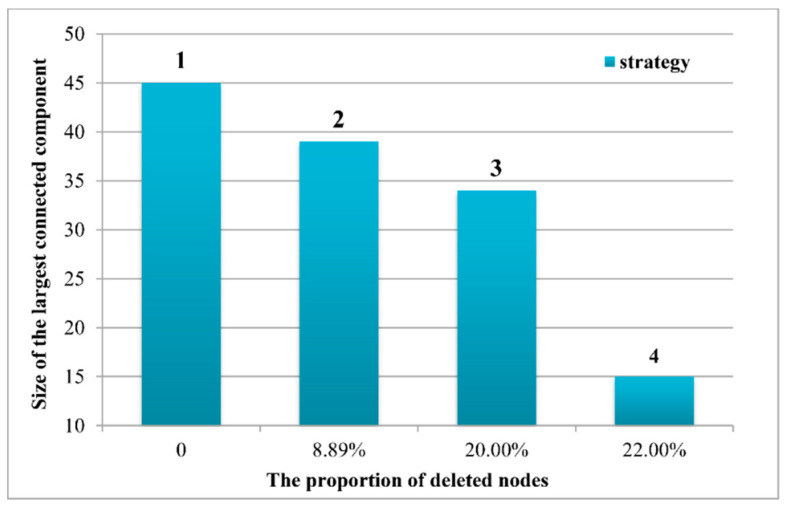
The sensitivity of nIFMA under different removal strategies.

To test the robustness of the interaction network and to evaluate the network dependence on specific nodes, we calculated the largest connected component size of the network under different removal strategies. Referring to the definition of the different types of cities in the Nationwide Urban System Planning of China 2006–2020, three strategies were confirmed: (1) The no removal strategy, which kept all of the cities in the network; (2) the national central city removal strategy, which removed all of the national central cities; and (3) the central city removal strategy, which removed all of the national and regional central cities. Considering the front results of the spatial characteristics analysis, we clearly observed the predominant role of Suzhou city (i.e., high *IFMA_i_*, high-high cluster with Shanghai in the YRD, a core role in the nIFMA and a high degree centrality), which was not the central city approved by the national planning. Thus, we set another strategy: (4) key city removal strategy, which removed all of the central cities and Suzhou. The results are shown in Figure 10.

Under the fourth removal strategy, the largest connected component size of the network gradually decreased. The sizes of the largest connected component under four different removal strategies were 45, 39, 35 and 15, and the linkage of the network had the strongest reduction under strategy (4). As can be observed, nIFMA was relatively robust. Specifically, the key role of Suzhou should be of more concern.

## 5. Discussion

### 5.1. Reasonability of the IFMA Index

To investigate the urban interaction features expressed by the information flow with geographical meaning in cyberspace, the IFMA index was constructed, and ESDA and CNA were employed to explore its characteristics in terms of spatial patterns and spatial networks. Depending on the construction of the IFMA indexes, we analyzed the rank and size of *IFMA_i_* to identify the focal cities that were more attractive for cyber information flow and justify the suitability of the IFMA index. Taking the *IFMA_i_* as an index to calculate the urban primary ratio of each city, the primary cities (i.e., Beijing and Shanghai) in the BTH and the YRD expressed obvious superiority compared to the other cities. The urban primary ratios were close to 2. Shenzhen and Guangzhou occupied the dominant positions in attracting public migration attention in the PRD; their urban primary ratios were relatively lower than that of the BTH and the YRD. Such a distribution pattern confirms with the previous researches, which analyzed the size and rank pattern of those regions based on the entities elements in geographical space [60,61,62,63,64]. It can be a representation of the reasonability of the IFMA index in describing urban features.

### 5.2. Interaction Features with Regional Positioning

From the perspective of the spatial pattern of urban interaction, the spatial autocorrelation of *IFMA_i_* in the three urban agglomerations was analyzed to further understand the spatial disparity of the inflow information derived from public migrant attention in cyberspace. The emergence of a High-High cluster pattern in each urban agglomeration highlighted that the clustering characteristic mainly belonged to the areas with high *IFMA_i_* values. The cities with relatively lower *IFMA_i_* were more inclined toward a random distribution. The high attention cities did not lead to a rise in the attention of their peripheral areas. This result may be caused by the continuous preference of migrant populations for central cities. According to the Report on China’s Migrant Population Development 2015, the central cities occupied 54.9% of interregional migrations until 2015. They were still the primary inflow destination of migrants, as demonstrated in the massive search attention in cyberspace. Currently, many central cities have begun to take steps to shunt the population and to divert migration to the peripheral areas, such as by raising the household threshold and transfer of industry. However, huge differences have not been fundamentally changed. The continuous low *IFMA_i_* of the surrounding area of central cities may be generated under such a situation, as displayed in Figure 6.

From the perspective of the spatial network, the network of *IFMA_ij_* based on the geographical spatial location and the graph theory was constructed to better interpret the spatial interaction characteristics of the cities. Basic network attributes, the urban degree centrality, the urban degree correlation and the robustness of the network have been measured. The following was found: (1) Through the exploration of basic network attributes, the small-world features of nIFMA can be identified. Closer intercity interactions and better network accessibility were shown to indicate the higher information transfer efficiency of nIFMA. (2) Based on the analysis of the urban degree centrality, it can be seen that the concentration of massive resources in geographical space triggers more migrant information flow in cyberspace. From the lens of individuals, the assembly of exotic investments, the introduction of talents, the preference of national policy, etc., can be a series of positive signals for more chances to improve individual life quality. The central cities that concentrate more regional resources demonstrated higher attractiveness than the regular cities. The urban characteristics presented by *IFMA_i_* were essentially harmonious with the actual urban population, economic, and urban status in regional planning from an urban network perspective. (3) Analyzing the results of the degree correlation, the disassortativity in the network could be proved by a negative degree correlation. Cities with a low degree centrality were inclined to pay more attention to cities with a higher degree centrality. The powerful attractiveness of national central cities and regional central cities, which was confirmed by national planning, was verified. As the one with urban political function of promoting regional balanced development and reducing interregional differences, central cities take more responsibility in linking diverse areas in the urban systems, which may attract more external expectations and concerns depicted by the growth of individual cyber concerns. (4) Through the diverse removal strategy of network nodes, the robustness of nIFMA was verified. The central cities played a significant role in the urban network, however, the network also had a strong ability to cope with an external attack on them.

Additionally, it is worth noting that the network role of parts of the cities did not completely meet the designed position of the national planning, typically exemplified by Shijiazhuang and Suzhou. Shijiazhuang is the provincial capital of Hebei Province and the only regional central city in the BTH, which should lead the development of regional network. However, the obvious marginalized location in the network, the relatively lower in-degree centrality among the central cities and other development indicators, such as GDP and population scale, all indicate the insufficient ability of Shijiazhuang to promote regional development. Focus on the Suzhou city where with the second largest economic aggregate and population scale in the YRD (even more than the capital city Nanjing), its networked influence can be found stronger than part of the regional central cities. A noticeable role of Suzhou in the YRD and even in the whole nIFMA, was identified. However, it has not been paid sufficient attention in terms of national policy and planning, which may be adverse to realizing its potential in guiding urban effective development. From this perspective, the regional policy should be modified to assist cities in boosting the efficiency of urban network construction and regional coordinated development, including building urban network interaction corridors with other regions, as well as eliminating development barriers derived from the national positioning of cities, etc.

### 5.3. Urban Interaction for Urban Spatial Reconstruction and Urban Life Improvement

Along with the rapid urbanization and industrialization, most cities in China have experienced the process of agglomeration and saturation. The suburbanization of large cities, the agglomeration of urban areas become the new evolution trend of cities. In such a context, spatial reconstruction of urban area is urgent for supporting the implementation of urban agglomeration economics. In 2014, the State Council of China approved the implementation of the National New-Type Urbanization Plan (2014–2020), which emphasizes the cities should be constructed in a balance and synergetic way. It symbolizes a departure from the sprawled construction of a single city to a form of linked and networked construction of multi cities. Urban interaction, the force linking individual cities into an organic urban system, become the key part in guiding China’s urbanization and spatial reconstruction. The interaction analysis based on the information flow in cyberspace displayed that, the core role of central cities in the three main urban agglomerations of China are prominent. Multi-center and networked develop pattern has merged but not been well developed in these areas. The interaction network has been built robustly, but the implementation of “the large cities lead the development of small city” proposed by the National New-Type Urbanization Plan is still insufficient. In the current stage of urban development, the reconstruction of urban space should be more systemic. Massive information interaction among cities signified the urgent need of cities for collaborative construction, such as the high *IFMA_ij_* between Shanghai and Suzhou, between Beijing and Shijiazhuang, etc. Based on such demand, the reconstruction of urban space should break the conventional framework of developing urban space surrounding the single urban center. More attention should be paid on the axis areas to construct fluent interaction passage, and support the collaborative development of cities based on the linked distribution of urban space.

The IFMA index, which was built based on public migrant attention, not only depicted the information flow between cities but also indicated the migrant potential of the population. Commonly, migration has been viewed as an approach to improve individual quality of life. The massive cyber migrant attention flow into the central cities may be a signal for the forthcoming migration and the need of improving the industrial, educational, and medical infrastructure of the central cities. We conduct the correlation analysis between *IFMA_i_* index and urban socio-economic index to estimate the related relationship between them. The results shown that, significant correlations can be observed between *IFMA_i_* with tertiary industrial output-value(0.87, *p* = 0.00), urban residents’ per capita disposable income(0.60, *p* = 0.00), participant rate of urban basic medical care system(0.51, *p* = 0.00) and the number of schools(0.75, *p* = 0.00). It indicated that higher economic profit and better public infrastructure are concerned by the potential migrants. The cities with higher IFMAi can moderately enhance the construction standard of urban infrastructure and industries to prepare for the subsequent migration. Besides, we conduct the correlation analysis between the IFMAi index with the unemployment rate (−0.15, *p* = 0.11) and per capita living area of cities (0.09, *p* = 0.28), no significant correlations imply that potential migrants take great exception for their future urban condition. The negative aspects of living in the central city have not been concerned. It is necessary to help the potential migrants comprehensively comprehending the life in central cities, to avoid the economical and psychical setback for migrants.

## 6. Conclusions

Urban interaction has immense potential to push the healthy development of urban areas in the current context of “space of flow”. In the internet era, the flow of information is becoming more and more frequent and now significantly denotes urban interaction relationships and urban network conditions. In this paper, we endeavor to identify urban interaction features expressed by the information flow in cyberspace, to extend the research angle of urban interaction and provide more references for the enacting of regional policy in the three main urban agglomerations of China. The IFMA index which derived from the public migrant attention has been constructed to quantify the information flow in cyberspace. Adopting the analysis framework coupling spatial pattern analysis and spatial network analysis, urban interaction features in the three urban agglomerations have been explored under the support of the IFMA index. The main conclusions are as follows: (1) The IFMA index presented a reasonable performance in depicting geographical features, which supported by similar results in the urban size and ranking analysis based on geographical entities. (2) the unconformity between urban role in the network and development positioning confirmed by national planning existed, the differentiated regional policy should be enacted to bring out the potential of cities to promote the healthy development of urban space. (3) in the context of new-type urbanization of China, urban interaction feature can be beneficial guidance for urban spatial reconstruction. Simultaneously, it also can provide some omen for the potential increase of migrants’ demand for urban infrastructure.

Urban areas are complex systems driven by diverse flows. This paper conducted an analysis of urban interaction features from the perspective of cyber information flows with geographical meaning. However, some limitations exist: First, in this paper, the cyber information flow was endowed with geographical meaning based on population migration. The exploration of information flow coupled with diverse meanings can be supplemented for elucidating more information regarding urban interactions. Second, the exploration in this paper was mainly conducted based on cross-section data; thus time-series data should be reinforced in future works to analyze dynamic interaction features.

## Figures and Tables

**Table 1:** Selection of the search keywords.

Migrant Reason	Keywords in Chinese	Translation in English
Work and trade	招聘, 租房	Recruitment, house renting
Study and training	学校	School
Relocation	房价, 地图, 天气	House price, map, weather

**Table 2:** Moran’s I of *IFMA_i_* in the different urban agglomerations.

Area	Moran’s I	E[I]	Sd	*Z*-Score	*p*-Value
**BTH**	0.04	−0.02	0.00	1.31	0.19
**YRD**	0.04	−0.03	0.01	0.70	0.49
**PRD**	0.36	−0.04	0.01	4.86	0.00

Note: BTH: the Beijing-Tianjin-105 Hebei metropolitan region; YRD: the Yangtze River Delta; PRD: the Pearl River Delta; Sd: standard deviation.

**Table 3:** The network indexes of the spatial network of *IFMA_ij_* (nIFMA).

Network		Node		Edge	
Degree	15.111	Number of nodes	45	Number of edges	342
Weighted degree	7.556	Average clustering coefficient	0.497	Average path length	1.642
Network diameter	3				
Graph density	0.172				
